# Comparison between non-spherical polyvinyl alcohol particles and tris-acryl gelatin microspheres after uterine artery embolization: a retrospective study

**DOI:** 10.25122/jml-2024-0327

**Published:** 2025-08

**Authors:** Mohammad Gharib Salehi, Mohammad Hossein Golezar, Arman Sijanivandi, Sana Delavari, Maryam Yeganegi, Shadi Nouri, Nazanin Farshchian

**Affiliations:** 1 Department of Radiology, Kermanshah University of Medical Sciences, Kermanshah, Iran; 2 Advanced Diagnostic and Interventional Radiology Research Center (ADIR), Medical Imaging Center, Tehran University of Medical Sciences, Tehran, Iran; 3 Clinical Research Development Unit, Ahvaz Jundishapur University of Medical Sciences, Ahvaz, Iran; 4 Department of Obstetrics and Gynecology, Iranshahr University of Medical Sciences, Iranshahr, Iran; 5 Department of Radiology, Arak University of Medical Sciences, Arak, Iran; 6 School of Medicine, Kermanshah University of Medical Sciences, Kermanshah, Iran

**Keywords:** leiomyoma, uterine artery embolization, polyvinyl alcohol, tris-acryl gelatin microspheres

## Abstract

The aim of this study was to compare leiomyoma infarction rates and embolization success using pelvic MRI following uterine artery embolization (UAE) using non-spherical polyvinyl-alcohol particles (nsPVA) or tris-acryl-gelatin microspheres (TAGM). A retrospective analysis was performed in 141 patients (mean age, 38 years) who underwent standard bilateral UAE with either nsPVA (*n* = 80) or TAGM (*n* = 61). Embolization success was defined as complete infarction of all discernible fibroids. Mann-Whitney U and independent-sample *t*-test were used to compare data types. A binary logistic regression was performed. 556 fibroids were evaluated, with a median uterine volume of 435 cm^3^ and a median dominant fibroid volume of 110 cm3. There were no significant differences between the two groups regarding baseline characteristics, including age (*P* = 0.446), uterine volume (*P* = 0.148), dominant myoma volume (*P* = 0.124), and non-infarcted myoma number (*P* = 0.092). The tumor infarction rate in the nsPVA and TAGM groups was 74% (251/337) and 79% (174/219), respectively, which was approximately similar (*P* = 0.191). Likewise, embolization success was similar among both groups (67.5% vs. 72.1% for nsPVA and TAGM, respectively, *P* = 0.589). There was an inverse relationship between the number of preliminary non-infarcted myomas and embolization success rate (*P* = 0.035). This study assessed the availability and side effects of these two substances, and patients underwent a 6-month follow-up MRI to evaluate possible consequences. According to post-embolization MRI, the leiomyoma infarction and embolization success rates for nsPVA and TAGM were similar. The decrease in uterine and myoma volumes was analogous to both drugs.

## INTRODUCTION

Uterine fibroids, or leiomyomas, are benign smooth muscle tumors of the uterus and are among the most common gynecological conditions encountered in clinical practice [1]. Patients with leiomyomas may present with menometrorrhagia, anemia, dysmenorrhea, or urinary and gastrointestinal symptoms resulting from the mass effect of an enlarged uterus [2].

Symptomatic fibroids have been treated by myomectomy or hysterectomy for years; however, over the past two decades, new treatment options, i.e., uterine artery embolization (UAE), have become available [3,4]. UAE is a safe, minimally invasive procedure for the treatment of uterine fibroids and is preferred over hysterectomy due to fewer complications, in addition to maintaining fertility status [5].

Several embolic agents have been introduced for UAE [6]. Among these, non-spherical polyvinyl alcohol particles (nsPVA) and tris-acryl gelatin microspheres (TAGM) are the most widely used and effective [7,8]. Comparing these agents has important clinical implications, as they may differ in terms of cost, ease of preparation, catheter-related complications, pain during injection, and potential side effects. Furthermore, differences in embolization success rates could directly influence patient outcomes.

Earlier studies suggested that spherical PVA was associated with lower infarction rates and poorer long-term outcomes compared with nsPVA or TAGM [9-12]. However, more recent work, including a randomized single-center trial of 60 patients, reported comparable results between spherical PVA (with larger particle size) and TAGM [13].

In comparative studies, TAGM has been associated with a higher clinical success rate and lower incidence of tumor enlargement, although no significant differences in adverse reactions or inflammatory responses were observed [14]. Tris-acryl microspheres, therefore, represent the preferred agent for UAE for uterine leiomyomas.

Nevertheless, in addition to safety and efficacy, the selection of the embolization agent depends on the cost, availability, and simplicity of preparation. Compared to PVA and TAGM, injection of the latter is more accessible and has a lower risk of catheter occlusion. Still, more vials of TAGM are required for the UAE while considering its higher price [15,16].

Residual fibroid enhancement on post-embolization MRI has been linked to reduced fibroid shrinkage, symptom recurrence, and higher reintervention rates. While some studies have examined each embolic agent individually, direct comparisons between nsPVA and TAGM in terms of post-embolization infarction and success remain limited. Prior work has suggested that factors such as uterine volume, fibroid volume, and fibroid location may influence the choice of embolic agent. However, most studies report no essential difference between nsPVA and TAGM with respect to short- and long-term imaging outcomes, symptom improvement, or reintervention rates [10,11,16,17].

In the present study, we compared leiomyoma infarction rates and embolization success using pelvic MRI following UAE performed with nsPVA or TAGM.

## MATERIAL AND METHODS

### Study design

This single-center retrospective observational study was approved by the Research Board and Ethics Committee of Kermanshah University of Medical Sciences. The study was conducted in accordance with the Declaration of Helsinki. Study details were explained to all patients, and written informed consent was obtained.

### Patients and data acquisition

**Figure 1:**
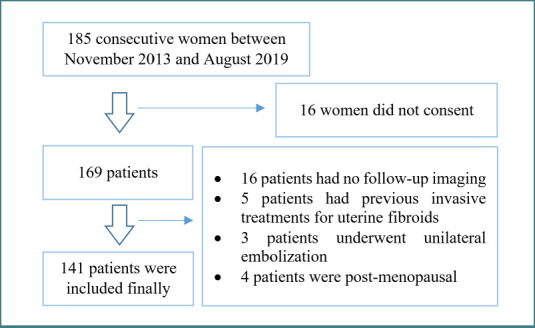
Flowchart of the study sample

The study included 185 Caucasian premenopausal women aged 25–50 years who underwent bilateral uterine artery embolization for leiomyomas between 2013 and 2019 at Imam Reza Hospital, Kermanshah, Iran. Eligibility required completion of both pre- and post-embolization contrast-enhanced pelvic MRI examinations. Patients were excluded if they had unilateral or incomplete UAE, prior myomectomy or UAE, or required reintervention during follow-up. Of 185 consecutive patients, 141 were eligible for analysis (mean age, 38 years). Data were extracted from medical records, and the final study sample is shown in Figure 1.

### Embolization procedure

All procedures were performed by an interventional radiologist with 15 years of experience, following a standardized bilateral UAE protocol used in previous studies [10,16]. The choice of embolic agent was determined by availability in the hospital pharmacy at the time of the procedure, not by patient characteristics or randomization.

After securing the left femoral artery access, the abdominal aorta was catheterized, and an initial pelvic aortography was performed to evaluate uterine artery collaterals and the dominant myoma blood supply. Then, the right iliac artery was catheterized, and a coaxial 3-F microcatheter was introduced. The microcatheter tip was placed at the transverse segment of the uterine artery. Uterine artery arteriography was obtained to evaluate myoma vasculature and confirm the correct catheter placement.

The uterine artery was embolized with either nsPVA (Contour; Boston Scientific, Marlborough, Massachusetts) or TAGM (Embosphere Microspheres; Merit Medical, South Jordan, Utah). A 355–500 µm nsPVA vial was used for the initial injection, and a 500–710 µm size range was selected for TAGM. The embolization endpoint for nsPVA was complete or near-complete stasis, with only minimal residual flow in the uterine artery after a 2 mL injection. The Waltman loop technique was used to catheterize the left uterine artery, and embolization was repeated using the same protocol. A final abdominal aortography was performed to assess for collateral flow. Significant ovarian artery flow was determined qualitatively at the discretion of the operator.

### Pre- and post-embolization imaging

Patients underwent pelvic contrast-enhanced MRI before and approximately 6 months after UAE using a 1.0-T imager (Intera, Philips Medical Systems, Best, The Netherlands). All images contained standard sequences: axial-sagittal-coronal T1 spin-echo (TR/TE 550-680/15- 25 msec), axial-sagittal T2 fast spin-echo (3475-4150/100-105), both for pre-contrast scans. Fat-suppressed T1 spin-echo sequences (TR/TE 480–500/7–10 msec) were acquired before and after intravenous injection of gadolinium-based contrast (0.1–0.2 mL/kg), with scans obtained 90–120 seconds post-injection. The field of view ranged from 25–38 cm, with a matrix of 256 × 256.

### MRI evaluation

An independent radiologist reviewed archived scans using a high-resolution digital display (IndoSurgicals, New Delhi, India). Baseline characteristics, including uterine volume, dominant myoma volume, and number of non-infarcted myomas, were recorded.

**Figure 2:**
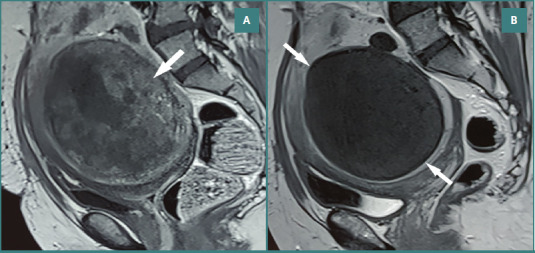
Uterine fibroid before and after uterine artery embolization. A, Pre-embolization sagittal contrast-enhanced T1-weighted MRI of the uterus showing approximately 50% enhancement of a large intramural myoma. B, Post-embolization sagittal contrast-enhanced T1-weighted MRI showing absence of enhancement consistent with complete infarction.

**Figure 3:**
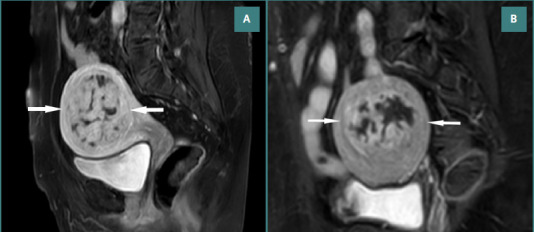
Pre-embolization enhancement of uterine fibroids on pelvic MRI. A, Sagittal fat-suppressed contrast-enhanced T1-weighted pelvic MRI demonstrating approximately 90% enhancement of a uterine fibroid. B, Sagittal fat-suppressed contrast-enhanced T1-weighted pelvic MRI showing nearly 80% enhancement of a uterine fibroid.

All visible sections of the myoma were evaluated to discern any vascularization. Fibroids that showed any enhancement were marked as non-infarcted. This value was estimated in the section where the largest diameter of the myoma had been depicted. In the case of multiple fibroids, the giant myoma was considered dominant, and the number of non-infarcted fibroids was counted for each patient. Representative MRI cases from Imam Reza Hospital are shown in Figures 2 (A-B) and 3 (A-B).

Follow-up MRIs were evaluated using the same protocol. Embolization success was defined as complete infarction of all visible fibroids. The embolization success and individual myoma infarction rate were calculated for each embolic agent. Mean changes in uterine volume, dominant fibroid volumes, and enhancement percentage were also calculated for each patient. A third radiologist tested the data validity.

### Statistical analysis

Data were analyzed using SPSS software (version 26.0; IBM Corp., Armonk, NY, USA). A 95% confidence interval was applied, and a two-tailed *P* < 0.05 was considered statistically significant. Normality of data distribution was assessed with the Shapiro–Wilk test. Continuous variables were analyzed using Mann–Whitney U or independent-samples *t* tests, as appropriate. Categorical variables were compared using the chi-square test or Fisher’s exact test. Infarction rate per fibroid and embolization success rate were compared between groups. Binary logistic regression was performed to assess the effect of pre-embolization factors on embolization success.

## RESULTS

A total of 556 uterine fibroids were assessed on archived MRI scans. Follow-up time was comparable between the nsPVA and TAGM groups (median, 6 months; range, 4–8 months; mean, 6.2 months for both; *P* = 0.745). There were no significant differences between the two groups in terms of baseline characteristics, including age (*P* = 0.446), uterine volume *(P* = 0.148), volume of dominant myoma *(P* = 0.124), and number of non-infarcted myomas (*P* = 0.092).

Comparison between the control and initial measurements of selected 35 patients showed no difference except for enhancement quantification of the dominant fibroid (median enhancement: 85% vs 75% for the reader and control radiologist, respectively; *P* = 0.032). However, mean enhancement change did not differ significantly between observers (85.7% ± 27.6% vs 86.9% ± 27.2%, *P* = 0.728). Due to this variability, enhancement values were excluded from subsequent analyses. The results are summarized in Table 1. Enhancement values were removed from the analysis owing to inter-observer variability. The results included initial values, and no consensus measurement was used.

**Table 1:** Measurements validity control

Characteristics	Reader	Control	*P* value
No. of patients	35	35	-
No. of baseline uninfarcted fibroids	108	108	-
No. of outcome uninfarcted fibroids	9	9	-
Baseline uterine volume (cm^3^) ^a^	377 (267-754)	440 (250-787)	0.545
Outcome uterine volume (cm^3^) ^a^	230 (134-440)	264 (183-411)	0.159
Baseline dominant fibroid volume (cm^3^) ^a^	110 (31-254)	112 (50-268)	0.646
Outcome dominant fibroid volume (cm^3^) ^a^	33 (19-118)	63 (19-110)	0.550
Baseline dominant fibroid enhancement (%) ^a^	85 (75-95)	75 (65-92.5)	0.032
Mean enhancement change (%) ^b^	85.7 ± 27.6	86.9 ± 27.2	0.728

a Median (25^th^ percentile-75^th^ percentile); ^b^Mean ± standard deviation)

Of 141 patients (80 treated with nsPVA, 61 with TAGM), 337 fibroids (61%) were embolized with nsPVA and 219 fibroids (39%) with TAGM. Baseline characteristics, including patient age, uterine volume, non-infarcted myoma numbers, dominant fibroid volume, and enhancement, were comparable across groups (Table 2).

**Table 2:** Baseline characteristics

Characteristics	Total	nsPVA ^a^ group	TAGM ^b^ group	*P* value
No. of patients	141	80	61	-
No. of total uninfarcted fibroids	556	337	219	-
Mean age (y) ^c^	37.7 ± 5.8	38.7 ± 6	36.7 ± 5.6	0.446
No. of uninfected fibroids per patient ^d^	3 (1-16)	3 (1-14)	2 (1-16)	0.092
Uterine volume (cm^3^) ^e^	435 (267-785)	496 (262-863)	419 (282-645)	0.148
Dominant fibroid volume^†^ (cm^3^) ^e^	110 (50-264)	137 (52-297)	94 (33-251)	0.122
Dominant fibroid enhancement^†^ (%) ^e^	85 (75-95)	85 (75-97.5)	85 (75-95)	0.675
Follow-up time^¶^ (m) ^d^	6 (4-8)	6 (4-8)	6 (4-8)	0.745

a non-spherical polyvinyl alcohol particles; ^b^tris-acryl gelatin microspheres; ^c^Mean ± standard deviation; ^d^Median (minimum-maximum); ^e^Median (25^th^ percentile-75^th^ percentile)

The embolization success rate was 67.5% (54/80) for nsPVA and 72.1% (44/61) for TAGM, with an overall success rate of 69.5% (98/141). The individual fibroid infarction rate was 74.5% (251/337) for nsPVA and 79.5% (174/219) for TAGM, with no statistically significant difference (*P* = 0.191). Overall, 76.4% (425/556) of fibroids were completely infarcted. Similarly, there was no difference between groups in other outcome measures, including mean enhancement change, uterine volume reduction, or dominant fibroid volume reduction (Table 3).

**Table 3:** Analysis of outcome variables

Characteristic	nsPVA ^a^ group	TAGM ^b^ group	*P* value
No. of total fibroids	337	219	-
Infarction rate per fibroid (%)	251/337 (74.5)	174/219 (79.5)	0.191
Embolization success rate (%)	54/80 (67.5)	44/61 (72.1)	0.598
Uterine volume change (%) ^c^	29.0 (54.3-1.1)	36.3 (58.4-21.2)	0.075
Dominant fibroid volume change (%) ^c^	48.2 (68.4-17.1)	49.2 (66.7-33.3)	0.091
Mean enhancement change (%) ^d^	84.6 ± 28.6	89.3 ± 23.2	0.108

a non-spherical polyvinyl alcohol particles; ^b^tris-acryl gelatin microspheres; ^c^Median (25^th^ percentile-75^th^ percentile); ^d^Mean ± standard deviation

Among 33 patients with a single fibroid (15 treated with nsPVA, 18 with TAGM), infarction rates were not significantly different (9/15 vs 15/18, *P* = 0.24).

The logistic regression model was significant (χ^2^ = 16.96, *P* = 0.030). Baseline age (*P* = 0.583), uterine volume (*P* = 0.277), and dominant fibroid size (*P* = 0.341) were not predictive of embolization success. However, the number of non-infarcted fibroids on pre-embolization MRI was inversely associated with embolization success (OR = 0.89; 95% CI, 0.79–0.99; *P* = 0.035). Thus, a greater number of non-infarcted fibroids at baseline was associated with lower success rates.

## DISCUSSION

Complete or near-complete infarction of all fibroids after uterine artery embolization on follow-up MRI is generally associated with favorable clinical outcomes [10,11,16,17]. Fibroid infarction rates after UAE with either nsPVA or TAGM have been reported as comparable [15,17-20], and our study supports this finding by demonstrating similar embolization success with both agents when assessed on individual fibroids.

Most published studies have focused on infarction of the dominant myoma, which is easier to evaluate. Czuczwar *et al*. assessed the effects of UAE with PVA on the volume of fibroids. They found that larger fibroids show a more predictable treatment response than smaller ones [21]. Similarly, Lacayo *et al*., in a study of 356 fibroids, found no difference between nsPVA and TAGM in individual infarction rates, consistent with our findings. Nevertheless, we did not use any predefined criteria, and they applied the uterus size criterion for choosing embolic agents, nsPVA for larger uteri, and TAGM for smaller ones, which led to an uneven population for each group [17].

From an imaging perspective, studies comparing UAE outcomes have shown no differences between nsPVA and TAGM regarding residual enhancement [11], reduction in enhancement percentage [10], dominant myoma infarction rate [16], or uterine and dominant fibroid volume reduction [11,16]. Our results confirm these observations. Spies *et al*. [16] also noted that while less nsPVA is required per procedure than TAGM, catheter occlusion is more likely with nsPVA. Thus, cost, availability, ease of injection, and technical performance are important considerations when selecting embolic agents.

A recent systematic review and meta-analysis by Sofy *et al*. reported that complete fibroid infarction was more likely with PVA than TAGM, whereas TAGM achieved superior results for partial infarction (<90%) at 24 hours. Both agents produced similar outcomes in terms of uterine and fibroid volume reduction and 90–99% infarction rates [15].

Our findings also suggest that patient age does not influence global infarction rates, consistent with reports by Naguib *et al*. [22], Spies *et al*. [23], and Lohle *et al*. [24], but in contrast to other studies [25,26] showing reduced volume shrinkage after UAE in older women.

Lower response to treatment in the older population can be explained by understanding the estrogen-related growth of a fibroid, which is induced by intra-lesional vasodilation. In the post-menopause state, a decrease in estrogen-related vasodilation inside the fibroid not only causes age-related fibroid shrinkage but also leads to a diminished response to embolization [21].

Age differences across study populations likely account for inconsistent findings. Our patients were predominantly of premenopausal age (ranging between 26 and 49 years), while most other studies with different results included patients older than 50 years, as well.

The present study did not find any correlation between predictive measures, including uterine and dominant myoma volume and embolization success, consistent with the findings of a similar project by Lacayo *et al*. [17]. They included 91 patients with a median age of 44 years who underwent UAE with either TAGM (77%) or nsPVA (23%) and found no correlation between uterine volume and global infarction rate. In contrast, some prospective studies [7,23] suggested that larger uterine and fibroid volumes predicted poorer embolization outcomes, while Czuczwar *et al*. [21] found larger fibroids to respond more favorably. Further research is needed to clarify these conflicting results.

An important finding of our study was the negative impact of the number of pre-embolization fibroids on embolization success: patients with more non-infarcted fibroids at baseline had lower success rates. Using a pre-determined dosage of an embolic agent may be worth reevaluating for multiple fibroids. However, this result contrasts with Lacayo *et al*. [13] and Lutfi *et al*. [27], who reported no correlation between fibroid number and treatment response, though their methods for assessing outcomes differed (volume reduction vs. enhancement reduction) and they used different embolic agents (nsPVA and Embozene Microspheres).

The choice of agent may be influenced by additional considerations even in cases when clinical outcomes show no difference. While both nsPVA and TAGM are widely available, TAGM is more expensive and requires more vials per procedure. TAGM is, however, easier to inject and less likely to obstruct catheters. In our study, PVA required greater dilution with saline and contrast to prevent clumping, but procedure times were not affected.

This study was retrospective, and embolic agent selection was based on availability rather than randomization, raising the possibility of selection bias. Although data collection and analysis were blinded, the interventional radiologist was aware of the embolic material used.

## CONCLUSION

Uterine artery embolization using non-spherical polyvinyl alcohol particles or tris-acryl gelatin microspheres had comparable individual myoma infarction rates determined on post-embolization contrast-enhanced pelvic MRI. In addition, both agents had similar rates of embolization success and uterine and myoma volume reduction. Finally, more research is recommended to investigate the clinical application and the difference between these factors.

## Data Availability

The data supporting these findings are available on request from the corresponding author. The data are not publicly available due to privacy or ethical restrictions.
